# Les tumeurs annexielles cutanées: étude anatomopathologique à propos de 96 cas

**DOI:** 10.11604/pamj.2015.20.389.6202

**Published:** 2015-04-20

**Authors:** Mohamed Réda El Ochi, Adil Boudhas, Mohammed Allaoui, Issam Rharrassi, Hafsa Chahdi, Abderrahman Al Bouzidi, Mohammed Oukabli

**Affiliations:** 1 Service d'Anatomie et de Cytologie Pathologique, Hôpital Militaire d'Instruction Mohammed V, Rabat, Maroc

**Keywords:** Tumeurs, annexes, peau, Tumors, Annexes, skin

## Abstract

Les tumeurs annexielles cutanées sont des tumeurs primitives cutanées à la fois rares et hétérogènes. Elles sont le plus souvent bénignes et rarement malignes. Elles sont dominées du point de vu morphologique par leur polymorphisme lésionnel. Le but de cette étude est de relever le profil épidémiologique et les différents aspects anatomopathologiques de ce groupe de tumeurs dans une cohorte de population marocaine et de les comparer avec les données de la littérature. Il s'agit d'une étude rétrospective de 96 cas de tumeurs annexielles cutanées colligées au service d'anatomie et de cytologie pathologique de l'Hôpital Militaire d'Instruction Mohammed V de rabat durant une période de 6 ans, de Janvier 2009 à Décembre 2014. Le pic de fréquence est situé entre 31 et 40 ans. L’âge moyen est de 36 ans avec une prédominance masculine (63,5%). Le siège de prédilection est la région de la tête et cou (47,9%). Les tumeurs bénignes (97,9%) sont plus fréquentes que les tumeurs malignes. La différenciation est folliculaire dans 51% des cas, eccrine/apocrine dans 44,8% des cas et sébacée dans 4,2% des cas. Le type histologique le plus fréquent est le pilomatrixome (33,4%) suivi par l'hidradénome (12,5%) et le spiradénome eccrine (11,5%). Les tumeurs annexielles cutanées sont rares et très variées. Le profil épidémiologique et les aspects anatomopathologiques qui ressortent sont globalement superposables à ceux rapportés dans la littérature. Elles sont majoritairement bénignes, à prédominance masculine et dominées par le pilomatrixome et l'hidradénome nodulaire. Les tumeurs malignes sont rares, agressives et surviennent à un âge plus avancé.

## Introduction

Les tumeurs annexielles cutanées (TAC) sont des tumeurs rares et très variées [1–3]. La classification de l'OMS de 2006 distingue 21 sous types de tumeurs bénignes et 15 sous types de tumeurs malignes [4]. Elles peuvent présenter une différenciation pilaire, eccrine, apocrine, sébacée et parfois mixte posant des problèmes diagnostiques [1, 5]. Ce diagnostic repose essentiellement sur l’étude anatomopathologique vu que l'aspect clinique n'est pas spécifique et parfois trompeur [6]. Les tumeurs bénignes sont les plus fréquentes; Les tumeurs malignes sont rares mais agressives et de pronostic péjoratif [1–3]. L'objectif de cette étude est de présenter une série de 96 cas de TAC pris en charge au sein du service d'anatomie et cytologie pathologique de l'Hôpital Militaire d'Instruction Mohammed V de Rabat, à fin de relever les différents aspects épidémiologiques et anatomopathologiques et de les comparer avec les données de la littérature.

## Méthodes

L’étude porte sur l'exploitation des fiches de liaison et des comptes rendus anatomopathologiques des TAC colligées au service d'anatomie et cytologie pathologique de l'Hôpital Militaire d'Instruction Mohammed V de Rabat sur une période de 6 ans entre janvier 2009 et décembre 2014. Dans notre série, nous avons exclu les hamartomes et les kystes annexiels et dysembryoplasiques qui sont des pseudo tumeurs et qui ne sont pas intégrés dans la classification de l'OMS de 2006. A partir des fiches de renseignement anatomopathologiques, nous avons collecté les données suivantes: l’âge, le sexe, la localisation, la taille de la tumeur et le type histologique.

## Résultats

Au total, 96 cas de TAC sont répertoriés entre janvier 2009 et décembre 2014.

### Caractères généraux de la population

**Figure 1:**
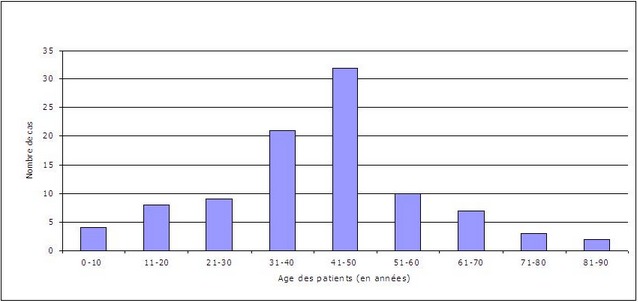
Répartition des cas selon l’âge

L’âge moyen est de 36 ans avec des extrêmes allant de 5 à 83 ans. L’étude de la répartition de la population selon l’âge (Figure 1) montre un pic de fréquence entre 31 ans et 40 ans (36,45%). La répartition de la population selon le sexe objective une prédominance masculine avec 61 cas (63,5%) contre 35 femmes (36,5%). Le sexe ratio est de 1,74.

### Résultats anatomopathologiques

**Figure 2:**
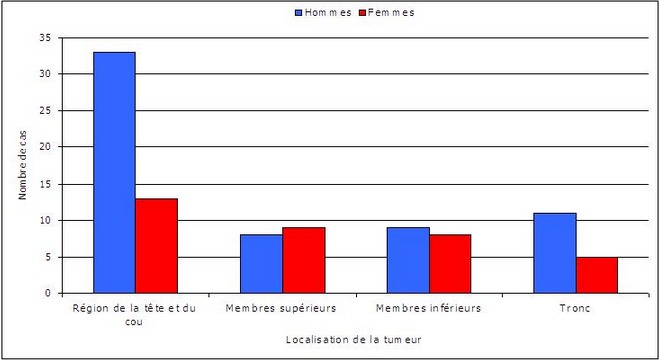
Localisation des tumeurs selon le sexe

**Figure 3:**
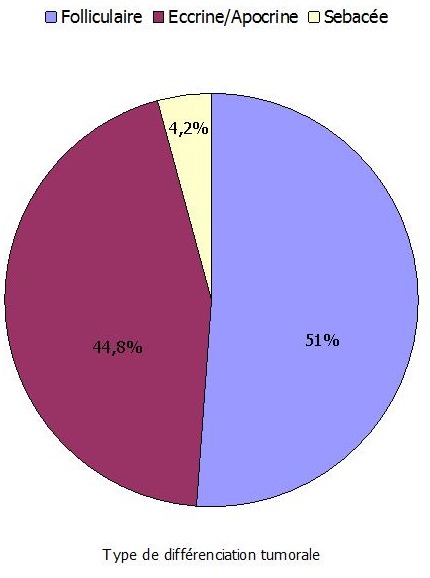
Différenciation tumorale

**Figure 4:**
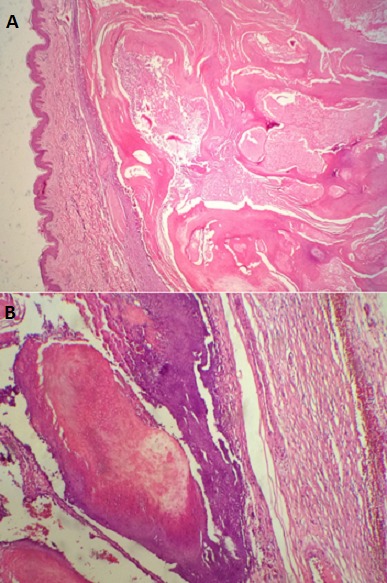
Pilomatrixome: A) derme siège d'une formation kystique remplie d'un matériel éosinophile (Gx40); B) paroi du kyste bordée par un épithélium pluristratifié basaloide avec présence de cellules momifiées dans la lumière (Gx100)

**Figure 5:**
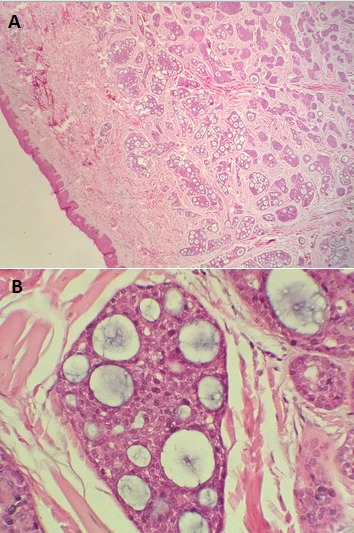
Cylindrome: A) revêtement cutané dont le derme abrite des lobules tumoraux arrondis (Gx40); B) lobules creusées de cavités contenant un matériel secretoire (Gx400)

Les tumeurs bénignes représentent 94 cas (97,9%) alors que les tumeurs malignes représentent 2 cas (2,1%). Sur les 96 cas, 46 (47,9%) sont localisés au niveau de la région de la tête et du cou contre 17 cas (17,7%) au niveau des membres supérieurs, 17 cas (17,7%) au niveau des membres inférieurs et 16 cas (16,7%) au niveau du tronc (Figure 2). Les 2 cas de tumeurs malignes recensés sont localisés au niveau de la joue et de la cuisse. Cette répartition est sexe indépendante (Figure 2). La différenciation tumorale est folliculaire dans 49 cas (51%), eccrine/apocrine dans 43 cas (44,8%) dont 2 tumeurs malignes et sébacée dans 4 cas (4,2%) (Figure 3). Parmi les tumeurs à différenciation folliculaire (Tableau 1), le type histologique prédominant est le pilomatrixome (Figure 4) retrouvé chez 32 cas (33,3%) suivi par le trichoépithéliome chez 5 cas (5,1%), le trichoadénome et le trichoblastome chez 4 cas chacun (4,2%) et le trichofolliculome et le trichilemmome chez 2 cas chacun (2,1%). Parmi les tumeurs à différenciation eccrine/apocrine, on note 12 cas (12,4%) d'hidradénome, 11 cas (11,4%) de spiradénome eccrine et 8 cas (8,3%) de syringome chondroide. Les autres types histologiques sont: l'hidrocytome (4 cas), le syringocystadénome papillifère (3 cas), le porome (2 cas), le cylindrome (1 cas) (Figure 5), le carcinome sudoral eccrine (1 cas) et le porocarcinome (1 cas). Dans la catégorie des tumeurs à différenciation sébacée, on retrouve uniquement l'adénome sébacé (4 cas). Dans la tranche d’âge de moins de 20 ans, le nombre de cas est de 12 réparti sur 3 types histologiques (tableau 2): dix pilomatrixome, un hidradénome et un porome eccrine (Tableau 2). Dans la tranche d’âge comprise entre 20 et 60 ans, le nombre de cas est de 72. Le pilomatrixome est le type histologique le plus fréquent retrouvé chez 20 cas, suivi par l'hidradénome chez 10 patients, le spiradénome eccrine chez 9 cas, ensuite le syringome chondroide chez 8 patients et l'adénome sébacé chez 4 patients. Les 21 malades restants sont mentionnés sur le tableau 2. Dans la tranche d’âge supérieure à 60 ans, le nombre de cas est de 12 dont 7 à différenciation folliculaire et 5 à différenciation sébacée (Tableau 2). La répartition des patients selon le sexe montre en général une prédominance masculine sauf pour l'hidradénome et le pilomatrixome (Tableau 3). En ce qui concerne la répartition des différents types histologiques selon la localisation, la région de la tête et du cou est le siège de prédilection pour toutes les catégories sauf l'hidradénome, le spiradénome eccrine et le porome (Tableau 4).

**Tableau 1:** Répartition globale des différents types histologiques

Différenciation tumorale	Types histologiques	Nombre de cas
**Folliculaire**	Pilomatrixome	32 (33,3%)
	Trichoépithéliome	5 (5,1%)
	Trichoblastome	4 (4,2%)
	Trichoadénome	4 (4,2%)
	Trichofolliculome	2 (2,1%)
	Trichilemmome	2 (2,1%)
**Eccrine/apocrine**	Hidradénome	12 (12,4%)
	Spiradénome eccrine	11 (11,4%)
	Syringome chondroide	8 (8,3%)
	Hidrocystome	4 (4,2%)
	Syringocystadénome papillifère	3 (3,1%)
	Porome	2 (2,1%)
	Cylindrome	1 (1,1%)
	Porocarcinome	1 (1,1%)
	Carcinome sudoral eccrine	1 (1,1%)
**Sébacée**	Adénome sebacé	4 (4,2%)

**Tableau 2:** Répartition des différents types histologiques selon l’âge

Différenciation tumorale	Type histologique	≤ 20 ans	20< âge ≤ 60 ans	> 60 ans	Total
**Folliculaire**	Pilomatrixome	10	20	2	32
	Trichoépithéliome	-	3	2	5
	Trichoadénome	-	3	1	4
	Trichoblastome	-	2	2	4
	Trichofolliculome	-	2	-	2
	Trichilemmome	-	2	-	2
**Eccrine/apocrine**	Hidradénome	1	10	1	12
	Spiradénome eccrine	-	9	2	11
	Syringome chondroide	-	8	-	8
	Hidrocystome	-	3	1	4
	Syringocystadénome papillifère	-	3	-	3
	Porome	1	1	-	2
	Cylindrome	-	-	1	1
	Porocarcinome	-	1	-	1
	Carcinome sudoral eccrine	-	1	-	1
**sébacée**	Adénome sébacé	-	4	-	4

**Tableau 3:** Répartition des types histologiques selon le sexe

Différenciation tumorale	Type histologique	Homme	Femmes	Total
**Folliculaire**	Pilomatrixome	12	20	32
	Trichoépithéliome	4	1	5
	Trichoadénome	3	1	4
	Trichoblastome	4	-	4
	Trichofolliculome	2	-	2
	Trichilemmome	2	-	2
**Eccrine/apocrine**	Hidradénome	5	7	12
	Spiradénome eccrine	9	2	11
	Syringome chondroide	6	2	8
	Hidrocystome	4	-	4
	Syringocystadénome papillifère	3	-	3
	Porome	2	-	2
	Cylindrome	1	-	1
	Porocarcinome	1	-	1
	Carcinome sudoral eccrine	-	1	1
**sébacée**	Adénome sébacé	3	1	4

**Tableau 4:** Répartition des différents types histologiques selon la localisation

Différenciation tumorale	Type histologique	Tête et cou	Membres supérieurs	Membres inférieurs	Tronc	Total
**Folliculaire**	Pilomatrixome	12	8	8	4	32
	Trichoépithéliome	4	-	-	1	5
	Trichoadénome	3	1	-	-	4
	Trichoblastome	4	-	-	-	4
	Trichofolliculome	2	-	-	-	2
	Trichilemmome	2	-	-	-	2
**Eccrine/apocrine**	Hidradénome	3	3	3	3	12
	Spiradénome eccrine	-	3	4	4	11
	Syringome chondroide	4	2	-	2	8
	Hidrocystome	3	-	-	1	4
	Syringocystadénome papillifère	2	-	1	-	3
	Porome	1	-	1	-	2
	Cylindrome	1	-	-	-	1
	Porocarcinome	1	-	-	-	1
	Carcinome sudoral eccrine	1	-	-	-	1
**sébacée**	Adénome sébacé	3	-	-	1	4

## Discussion

Les TAC sont des tumeurs rares et très variées [1–3]. Elles sont le plus souvent bénignes et rarement malignes [6, 7]. Peu d’études se sont intéressées à ce groupe de tumeurs [7]. Notre étude porte sur une série de 96 cas de TAC dont le profil épidémiologique et l'aspect anatomopathologique sont résumés dans le Tableau 5 et comparés à ceux de la littérature.

**Tableau 5:** Résultats rapportés selon les séries

		Rajalakshmi et al. [6]	Sharma et al. [11]	Nair [12]	Notre série
Nombre de cas		21	56	33	96
Pic de fréquence		-	51- 60 ans	11- 20 ans	31- 40 ans
Localisation prédominante		Tête et cou47,6%	Tête et cou64,28%	Tête et cou46%	Tête et cou47,9%
Pourcentage des tumeurs bénignes		90,48%	80,36%	100%	97,9%
Différenciation tumorale	Eccrine/apocrine	61,90%	42,86%	57,65%	44,80%
	Folliculaire	33,34%	35,71%	36,36%	51%
	Sébacée	4,76%	21,43%	6,06%	4,2%
Types histologiques prédominants		Pilomatrixome	Hidradénome	Syringome	Pilomatrixome
		Hidradénome	Pilomatrixome	Trichoépithéliome	Hidradénome
Nombre de cas de tumeurs malignes		2	11	0	2

### Répartition selon l’âge

L’âge moyen dans notre série est de 36 ans. Il est comparable à celui rapporté par Gayathri et al. (35,2%) et Samaila et al. (33 ans) [8, 9]. En ce qui concerne le pic de fréquence, on note une divergence entre les auteurs. Pour notre série et pour Radhika et al. et Kambiz et al., il est situé à la troisième décade [7, 10]. Pour d'autre, il est à la cinquième décade voire à la deuxième [11, 12].

### Répartition selon le sexe

Pour certains auteurs [6, 11], il existe une légère prédominance masculine, alors que pour Nair et al. et Saha et al., le sexe ratio est de 1:2,3 et 1:1,88 respectivement [12, 13]. Dans notre série on note une nette prédominance masculine (sexe ration de 1,74:1) due vraisemblablement à la prédominance du sexe masculin en milieu militaire.

### Répartition selon la localisation

Les TAC sont essentiellement localisées au niveau de la région de la tête et du cou tandis que les membres et le tronc sont moins touchés [6, 11, 12]. Ceci est dû à la grande abondance des structures annexielles cutanées à ce niveau. Pour Sharma et al. [11], les TAC touchent la région de la tête et du cou dans 64,28% des cas, le tronc dans 14,28% des cas et les membres supérieurs dans 12,5% des cas. Ceci est vrai également pour Nair [12] et pour notre série avec une localisation au niveau de région de la tête et du cou dans 46% et 47,9% respectivement.

### Répartition selon le caractère bénin ou malin

Dans notre série, les tumeurs bénignes sont les plus fréquentes. Ceci est en accord avec toutes les autres statistiques de la littérature [6, 8, 9, 11, 12]. Les tumeurs malignes restent rares et surviennent à un âge plus avancé [6, 12].

### Répartition selon la différenciation tumorale

Pour Nair et Ankit et al., les tumeurs eccrine/apocrine sont les plus fréquentes suivies par les tumeurs folliculaires et sébacées [11, 12] Dans cette étude, on relève que la différenciation folliculaire est la plus fréquente (51%), suivie par les tumeurs eccrines/apocrines (44,8%) et les tumeurs sébacées (4,2%). Ceci est dû à la grande fréquence des pilomatrixomes dans notre série. Ce dernier est le type histologique le plus fréquent suivi par l'hidradénome [6, 7]. Les autres sous types histologiques sont plus rares [6, 7].

## Conclusion

Ce travail permet de rapporter le profil épidémiologique et les différents aspects anatomopathologiques des TAC prises en charge dans notre formation durant une période de 6 ans (2009-2014). Le pic de fréquence est situé entre 30 et 40 ans avec une prédominance masculine (63,5%). Les tumeurs bénignes (97,7%) sont beaucoup plus fréquentes que les tumeurs malignes. La région de la tête et du cou est le siège de prédilection. Les types histologiques les plus couramment rencontrés sont le pilomatrixome et l'hidradénome.

## References

[1] Elder D, Elinistas R, Ragsdale BD, Elder D, Elinistas R, Jaworsky C, Johnson B (1997). Levers Histopathology of the skin 8 th ed: Tumours of the epidermal appendages.

[2] Obaidat NA, Alsaad KO, Ghazarian D (2007). Skin adnexal neoplasms-part 1: An approach to tumours of the pilosebaceous unit. J Clin Pathol..

[3] Obaidat NA, Alsaad KO, Ghazarian D (2007). Skin adnexal neoplasm-part 2: An approach to tumours of cutaneous sweatglands. J Clin Pathol..

[4] Le Boit PE, Burg G, Weedon D, Sarasain A (2006). World Health Organization Classification of Tumours: Pathology and Genetics of Skin Tumours.

[5] Storm CA, Seykora JT (2002). Cutaneous adnexal neoplasms. Am J Clin Pathol..

[6] Rajalakshmi V, Selvakumar S, Rajeswari K, Meenakshisundaram K, Veena G, Ramachandran P (2014). Case series of skin adnexal tumours. J Clin Diagn Res..

[7] Kamyab-Hesari K, Balighi K, Afshar N, Aghazadeh N, Rahbar Z, Seraj M, Rayati M (2013). Clinicopathological study of 1016 consecutive adnexal skin tumors. Acta Med Iran..

[8] Gayathri SS, Ezhilvizhi A, Ashok kumar S (2012). An analysis of skin appendageal tumours in South India. J of evol and den sci..

[9] Samaila M (2008). Adnexal skin tumors in Zaria, Nigeria. Ann Afr Med..

[10] Radhika K, Phaneendra BV, Rukmangadha N, Reddy MK (2013). A study of biopsy Confirmed skin adnexal tumours: experience at a tertiary care teaching hospital. J Clin Sci Res..

[11] Sharma A, Paricharak DG, Nigam JS, Rewri S, Soni BB, Omhare A, Sekar S (2014). Histopathological Study of Skin Adnexal Tumours-Institutional Study in South India. J Skin Cancer..

[12] Nair PS (2008). A clinicopathologic study of skin appendageal tumors. Indian J Dermatol Venereol Leprol..

[13] Saha A, Das NK, Gharami RC, Chowdhury SN, Datta PK (2011). A clinico-histopathological study of appendageal skin tumors, affecting head and neck region in patients attending the dermatology opd of a tertiary care centre in eastern India. Indian Journal of Dermatology..

